# Predictive value of circulating plasma mitochondrial DNA for Sepsis in the emergency department: observational study based on the Sepsis-3 definition

**DOI:** 10.1186/s12873-020-00320-3

**Published:** 2020-04-16

**Authors:** Lifeng Wang, Wei Zhou, Kaiwen Wang, Shuangjun He, Yi Chen

**Affiliations:** 1 Department of Emergency, South Campus, Renji Hospital, School of Medicine, Shanghai Jiao Tong University, Shanghai, China; 2 Department of Rheumatology, South Campus, Renji Hospital, School of Medicine, Shanghai Jiao Tong University, Shanghai, China

**Keywords:** Mitochondrial DNA, Lactate, Sepsis, Septic shock, Predictive value

## Abstract

**Background:**

The definition of sepsis is regularly updated; however, there is no standard diagnostic test. To improve diagnosis and prognostic prediction, the aim of this study was to determine the predictive value of circulating plasma mitochondrial DNA (mtDNA) levels in patients admitted to the emergency department (ED) with sepsis.

**Methods:**

A total of 107 patients hospitalized from June 2018 to January 2019 were divided into the sepsis (*n* = 72) and septic shock (*n* = 35) groups based on the sepsis-3 definition. Clinical and laboratory data were measured within 24 h of admission. The mtDNA concentrations in clarified plasma were estimated by quantitative polymerase chain reaction. Binary logistic regression analysis and the receiver operating characteristic (ROC) curve were used to determine predictive value of mtDNA and other markers for sepsis outcome (28-day mortality).

**Results:**

The median plasma mtDNA levels on admission were significantly higher in the septic shock patients than in the sepsis patients (134,252(IQR 70215–203,184) vs. 59,945(IQR 13274–95,319) copies/μL, *P* < 0.01), and were also higher in non-survivors than in survivors within 28 days (165,291(IQR 89919–272,228)vs. 63,025(IQR 17031–98,401)copies/μL, *P* < 0.01). Binary logistic regression showed that plasma lactate and mtDNA levels were independent risk factors for 28-day mortality [odds ratio (OR) 1.341, 95% confidence interval (CI) 1.035–1.736, *P* = 0.026 and OR 13.299, 95%CI 2.765–63.956, *P* = 0.001, respectively). The area under the ROC curve values for plasma mtDNA levels, lactate concentration, and their combined were 0.781 (*p* < 0.001, 95%CI 0.671–0.891), 0.733 (*p* < 0.001, 95%CI 0.635–0.832), and 0.799 (*p* < 0.001, 95%CI 0.698–0.901), respectively. The calibration test for the combined variable showed X^2^ of 2.559 and *P* = 0.923.

**Conclusion:**

A higher plasma mtDNA level was associated with a poor prognosis of sepsis in the emergency room, and could serve as a predictor of sepsis for 28-day mortality.

## Background

The updated definition of sepsis is a life-threatening organ dysfunction caused by a dysregulated host response to infection [1], and remains a common and lethal syndrome. Although outcomes have improved, sepsis-related mortality is still higher than 25–30%, and reaches up to 40–50% in cases of septic shock as a subset of sepsis with particularly profound circulatory, cellular, and metabolic abnormalities [1, 2]. Sepsis is a major public health concern, accounting for more than $20 billion (5.2%) of total US hospital costs in 2011 [3]., Moreover, the reported incidence of sepsis is increasing [4, 5], likely reflecting the aging population with more comorbidities, greater recognition of the condition [6], and, in some countries, reimbursement-favorable coding [7]. There is currently no gold-standard diagnostic test of sepsis or septic shock. Although monitoring of lactate levels is used as a prognostic guide or indicator of illness severity, the value of this marker is highly controversial. Some suggested that elevated lactate levels represent an important marker of “cryptic shock” in the absence of hypotension. Others voiced concern about its specificity and that the non-availability of lactate measurement in resource-poor settings would preclude a diagnosis of septic shock [1]. Lactate level is a sensitive, albeit nonspecific, stand-alone indicator of cellular or metabolic stress rather than “shock” [8] .Therefore, there is an urgent need to find a specific factor that can accurately predict the severity and mortality risk of sepsis.

Mitochondrial DNA (mtDNA) has many similarities with bacterial DNA because of their shared common ancestry. Accordingly, increasing evidence points to mtDNA as a danger signal that is recognized by the innate immune system to directly modulate the inflammatory response [9]; thus, extracellular mtDNA can activate signaling pathways and promulgate inflammation [10]. This raises the possibility that mtDNA may serve as a surrogate of disease severity or even a predictor of mortality in critically ill patients. In a seminal series of studies, Zhang et al. [11–13] demonstrated that patients admitted with trauma showed significant elevations of mtDNA concentrations in the plasma and injured tissues. These results were validated in a rat model of trauma/hemorrhagic shock in which the plasma mtDNA levels were elevated for 7 days after injury [11]. Nakahira et al. [14] further demonstrated that an elevated mtDNA level was associated with intensive care unit (ICU) mortality among multiple cohorts, and could improve risk prediction in the field of critical care illness.

Thus, both basic research and clinical trials have now provided clear evidence that circulating mtDNA should be considered a new subtype of a damage-associated molecular pattern (DAMP) that is elevated in critically ill diseases such as sepsis, trauma, or hemorrhagic shock. However, data regarding the role of plasma mtDNA in adult sepsis is conflicting, and the clinical ramifications of this finding remain elusive. Moreover, clinical trials provide conflicting evidence about the association of mtDNA with mortality in critically ill patients [15], and the consensus definition and clinical criteria for sepsis change every few years. Since some recent studies have been performed on the basis of the newest criteria sepsis-3, we used this consensus definition in the present study to determine the clinical value of plasma mtDNA levels as a biomarker for predicting the mortality of sepsis in patients at the emergency department (ED) of a Chinese hospital.

## Methods

### Study design, setting, and population

This observational study was conducted in the ED of Shanghai South Campus, Renji Hospital, which is an urban university tertiary-care hospital with approximately120,000 ED visitors per year. From June 2018 to January 2019, 116 consecutive non-traumatic patients who fulfilled the sepsis-3 criteria defined by the American College of Chest Physicians/Society of Critical Care Medicine (ACCP/SCCM) were enrolled in the study. The exclusion criteria were as follows: younger than 18 years old, terminal stage of disease (malignant cancer of any type), end-stage renal disease, and patients who declined to participate in the study themselves or via their relatives. Ultimately, 107 consecutive patients were enrolled, and nine patients were excluded. All procedures performed in studies involving human participants were in accordance with the ethical standards of the Shanghai Jiaotong University School of Medicine, Renji Hospital Ethics Committee (NO.2016-109 k) and with the 1964 Helsinki declaration and its later amendments or comparable ethical standards. All of the patients provided written informed consent.

### Definitions and grouping

The patients were divided into a sepsis group and a septic shock group according to the sepsis-3 criteria [1]. That is, sepsis was clinically identified as an infection with a SOFA score ≥ 2, and patients with septic shock were clinically identified according to vasopressor requirement to maintain a mean arterial pressure of 65 mmHg or greater and a serum lactate level > 2 mmol/L (> 18 mg/dL) in the absence of hypovolemia.

Septic cardiomyopathy (SCM) was defined as an acute syndrome of cardiac dysfunction that is unrelated to cardiac ischemia in patients with sepsis [16], B-type natriuretic peptide (BNP) and troponin elevations appear to reflect SCM.

### Preparation and quantification of plasma DNA

Blood samples were drawn from patients in the two groups within 24 h after admission, transferred into ethylenediaminetetraacetic acid-coated blood collection tubes, and processed within 2 h after venipuncture [14]. The samples were left to rest for 30 min and then centrifuged immediately at 1914×*g* for 10 min to separate the plasma from the cellular components. The plasma samples were stored at − 80 °C for batch analysis.

DNA was isolated from the plasma using the QIAamp Blood Mini Kit (#51106; Qiagen GmbH, Hilden, Germany) according to the manufacturer’s manual. All samples were thawed on ice, and the level of the mtDNA gene cytochrome b was measured by a SYBR Green dye-based quantitative real-time polymerase chain reaction (qPCR) assay (TaKaRa, Japan) using an ABI Prism7900HT detection system with the following primers: forward, ATGACCCCAATACGCAAAAT; reverse, CGAAGTTTCATCATGCGGAG. The PCR mixture was set up in a reaction volume of 10 μL using 5 μL of 2× SYBR green Master Mix (2×), 0.5 μL forward primer (1 μM), 0.5 μL reverse primer (1 μM), 3 μL of nuclease-free H_2_O, and 1 μL of plasma extract. The following thermocycler conditions were used: 3-min incubation at 95 °C, followed by 40 cycles of initial denaturation at 95 °C for 30 s, annealing at 54 °C for 45 s, and elongation at 68 °C for 1 min. The concentration of mtDNA was determined using a standard curve generated by qPCR with construct plasmids (pMD® 18-T Vector; Sangon Biotech, Shanghai) containing the human mitochondrial cytochrome B gene sequences described above. Calibrators were prepared by serial 10-times dilution of the stock solution and contained 10^2^ to 10^8^ mtDNA copies/μL.

### Statistical analysis

Clinical and laboratory data are expressed as number (percent) or median [interquartile range (IQR), i.e., 25th–75th percentile], as appropriate. Statistical calculations were performed with IBM SPSS software version 26.0 (SPSS Inc., Chicago, IL, USA). Bivariate comparisons were conducted with the Mann-Whitney U test for continuous variables, and with the chi-squared test or Fisher’s exact tests for categorical variables. Bonferroni adjustment was adopted to select variables (treatment process and sepsis indused organ injury werec excluded). The influence of clinical and baseline laboratory data, including respiratory tract infection (RSTI),coronary heart disease (CHD), in addition to the log mtDNA concentration and lactate levels (which adjusted *P* <0.002), on 28-day mortality was evaluated in bivariate logistic regression models to determine independent predictors. Receiver operating characteristic (ROC) curve analyses were used to assess the predictive value of mtDNA on mortality by Sigma Plot 14.0, and cut-off values were calculated according to Youden’s index. The sensitivities and specificities were further used to calculate the positive and negative likelihood ratios. The Hosmer-Lemeshow goodness of fit test was used for verifying model calibration. All statistical tests were two-tailed, and *P* < 0.05 was considered to indicate a statistically significant difference.

## Results

The baseline characteristics of the sepsis (*n* = 72) and septic shock group (*n* = 35) on admission to the ED are presented in Table 1. The median age for the septic shock patients was 73 (64–83) years, and 60% of the patients were male. Similarly, the median age for the septic patients was 68 (56.25–80) years, and 59.72% were male. There was no significant difference in underlying chronic medical problems between the two groups at the time of enrollment except for CHD (*P* < 0.01). There was a significant difference in the primary site of infection, including respiratory tract infection (*P* < 0.001) and urinary tract infection (*P* = 0.014) between the two groups. Clinical parameters such as CRRT (*P* = 0.002), requirement of mechanical ventilation (*P* < 0.001), vasopressor use (*P* < 0.001), and SOFA score (*P* < 0.001) were all significantly higher in the septic shock group. There was no significant difference in peripheral blood risk factors such as white blood cell count, hemoglobin, platelet count, C-reactive protein (CRP), and procalcitonin (PCT) between the two groups. The median mtDNA level and median lactate concentration were significantly higher in the septic shock patients than those of the sepsis patients on admission (*P* = 0.001 and *P* < 0.0001, respectively). The plasma mtDNA concentration did not correlate to the commonly used acute-phase markers CRP and PCT (γ = − 0.001, *P* = 0.994 and γ = − 0.145, *P* = 0.141, respectively), but was correlated with the SOFA score (γ = 0.344, *P* < 0.001).

**Table 1:** Baseline characteristics of the ED patients

Number (Percent) or Median (Interquartile Range)						
Variable	Sepsis (*n* = 72)	Septic shock (*n* = 35)	*P* value	survivors (*n* = 81)	non-survivors (*n* = 26)	*P* value
Age (Years)	68(56.25–80)	73(64–83)	0.27	68(55.5–79)	67.25(78.5–84.25)	0.014
Gender (Males) Underlying diseases	43(59.72%)	21(60%)	0.98	48(59.3%)	21(60%)	1.00
Hypertension	25(34.7%)	16(45.7%)	0.296	26(32.1%)	15(57.7%)	0.036
Diabetes mellitus	20(27.8%)	10(28.6%)	1.00	22(27.2%)	8(30.8%)	0.803
Coronary heart disease	10(13.9%)	17(48.6%)	<0.001	12(14.8%)	15(57.7%)	<0.001
cerebral infarction	12(16.7%)	11(31.4%)	0.131	15(18.5%)	8(30.8%)	0.271
Chronic pulmonary disease	6(8.3%)	5(14.3%)	0.498	5(6.2%)	6(23.1%)	0.023
Autoimmune disease	7(9.7%)	5(14.3%)	0.522	7(8.6%)	5(19.2%)	0.159
Previous surgical history	10(13.9%)	3(8.6%)	0.539	11(13.6%)	2(7.7%)	0.730
Infection site						
Respiratory tract infection	29(40.3%)	28(80%)	<0.001	34(42%)	23(88.5%)	<0.001
Urinary tract infection	22(30.6%)	3(8.6%)	0.014	24(29.6%)	1(3.8%)	0.007
Gastrointestinal infection	7(9.7%)	3(8.6%)	1.00	9(11.1%)	1(3.8%)	0.445
Hepatobiliary system infection	7(9.7%)	4(11.4%)	0.747	9(11.1%)	2(7.7%)	1.000
skin infection	4(5.6%)	4(11.4%)	0.434	5(6.2%)	3(11.5%)	0.399
intracranial infection	0	1(2.9%)	0.327	1(1.2%)	0	1.000
Unknown origin	8(11.1%)	1(2.9%)	0.266	8(9.9%)	1(3.8%)	0.449
Bloodstream infection	21(29.2%)	7(20%)	0.357	24(29.6%)	4(15.4)	0.202
CRRT	1(1.4%)	7(20%)	0.002	8(9.9%)	5(19.2%)	0.002
Mechanical ventilation	3(4.2%)	23(65.7%)	<0.001	8(9.9%)	18(69.2%)	<0.001
Vasopressor use	6(8.3%)	29(82.9%)	<0.001	14(17.3%)	21(80.8%)	<0.001
SOFA	2 (2–3)	10 (8–12)	<0.001	2(2–4)	9(7–12.25)	<0.001
AKI	29(40.3%)	25(71.4%)	0.004	37(45.7%)	17(65.4%)	0.114
SCM	16(22.2%)	24(68.6%)	<0.001	21(25.9%)	19(73.1%)	<0.001
WBC(^a^10^9^/L)	10.67(6.74–15.49)	12.62(8.65–14.77)	0.150	10.59(6.74–15.33)	13.01(11.36–14.87)	0.028
Hb(g/L)	122(111–135.75)	119(94–139)	0.834	125(111.5–137)	106(81.75–138.25)	0.062
Plt(^a^10^9^/L)	177(122.75–225.75)	149(71–210)	0.449	152(110–220)	187.5(78.5–235)	0.237
PCT (ng/ml)	4.24(0.90–13.35)	7.21(1.09–45.42)	0.632	4.63(1.02–15.59)	2.31(0.79–27.64)	0.865
CRP (mg/L)	139.49(72.50–200)	133.82(41.69–200)	0.835	142.43(68.97–200)	118.29(53.18–189.76)	0.647
Lactate (mmol/L)	1.90(1.40–2.80)	2.80(2.30–5.42)	<0.001	1.96(1.40–2.98)	2.75(2.38–4.78)	0.001
mtDNA (copies/ul)	59,945(13274–95,319)	134,252(70215–203,184)	0.001	63,025(17031–98,401)	165,291(89919–272,228)	0.001
28-d mortality	2(2.8%)	24(68.6%)	<0.001			
90-d mortality	5(6.9%)	26(74.3%)	<0.001			

^a^:CRRT, continuous renal replacement therapy; *SOFA* sequential organ failure assessment; *WBC* white blood cell; *Hb* hemoglobin; *Plt* platelet; *PCT* procalcitonin; *CRP* C-reactive protein; *AKI* acute kidney injury; *SCM*, septic cardiomyopathy

**Fig. 1:**
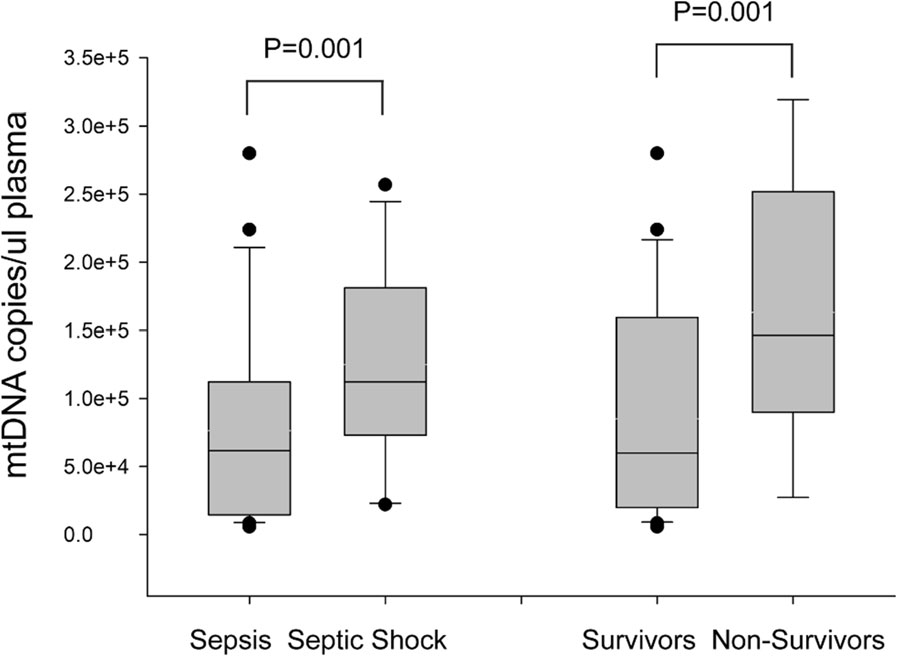
Plasma mtDNA levels in sepsis /septic-shock group and survivors/non-survivors group

In addition, the overall 28-d (*P* < 0.001) and 90-d (*P* < 0.001) mortality rates were significantly higher in the septic shock patients. To determine the predictive value of plasma mtDNA and other variables following sepsis, we compared their levels between survivors and non-survivors. As shown in Table 1, the median plasma mtDNA and lactate levels in non-survivors were significantly higher than those of the survivors (both *P* = 0.001). Box plots of plasma levels of mtDNA in the sepsis/septic-shock group and survivors/non-survivors group are shown in Fig. 1. Univariate analysis (Table 2) indicated a difference in clinical and baseline characteristics between the survivors and non-survivors, including respiratory tract infection (RSTI)(P<0.001) CHD (*P* < 0.001)Lac(*P* = 0.019) and Log mtDNA (*P*<0.001). These significant variables were then included in the bivariate logistic regression model, demonstrating that lactate and log mtDNA levels were independent predictors of survival (*P* = 0.017 and *P* = 0.003, respectively; Table 2).

**Table 2:** The binary logistic regression of risk factors for 28-d mortality

Variable	Univariate	analysis		Multivariate	analysis	
	OR*	95%CI*	*P* value	OR	95%CI	*P* value
RSTI*	10.598	2.942–38.174	<0.001	13.711	1.927–97.545	0.009
CHD*	7.841	2.912–21.113	<0.001	5.749	1.649–20.04	0.006
Lac*	1.228	1.035–1.457	0.019	1.313	1.051–1.64	0.017
Log mtDNA*	11.892	3.141–45.027	<0.001	10.352	2.205–48.609	0.003

*: *OR* Odds ratio; *95% CI* 95% Confidence interval; *RSTI* Respiratory tract infection; *CHD* Coronary heart disease; *Lac* Lactate; Log mtDNA, Log _10_^mtDNA^

**Fig. 2:**
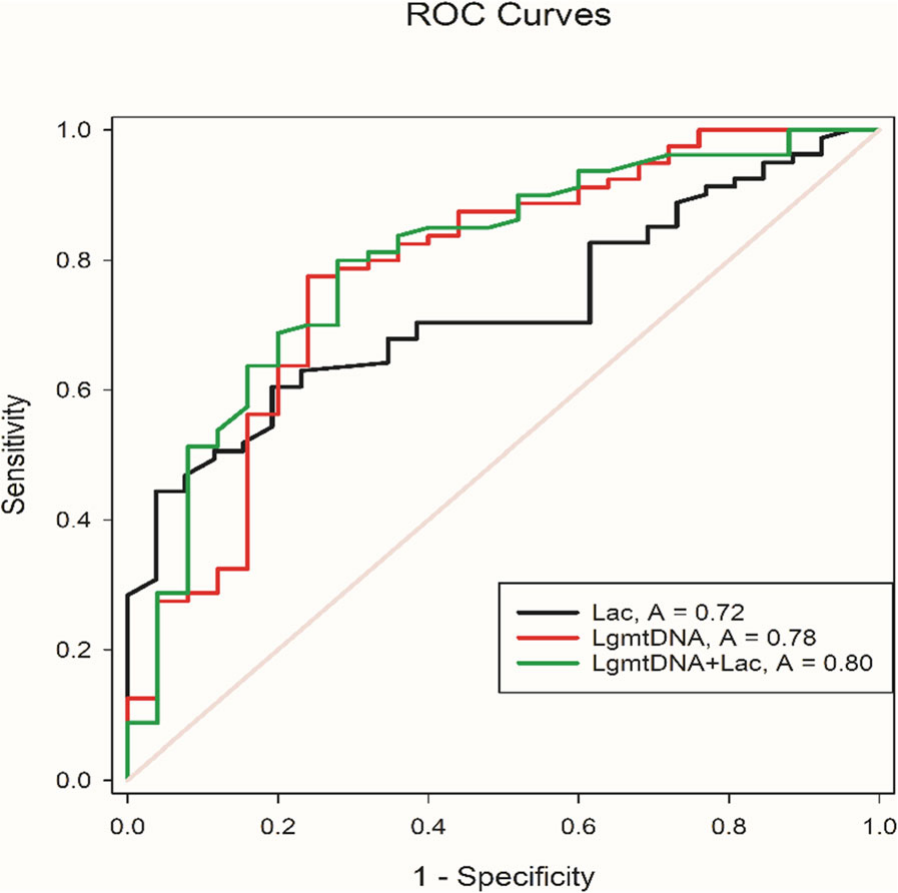
ROC curves of Lac, Log mtDNA and Log mtDNA+ Lac

**Fig. 3:**
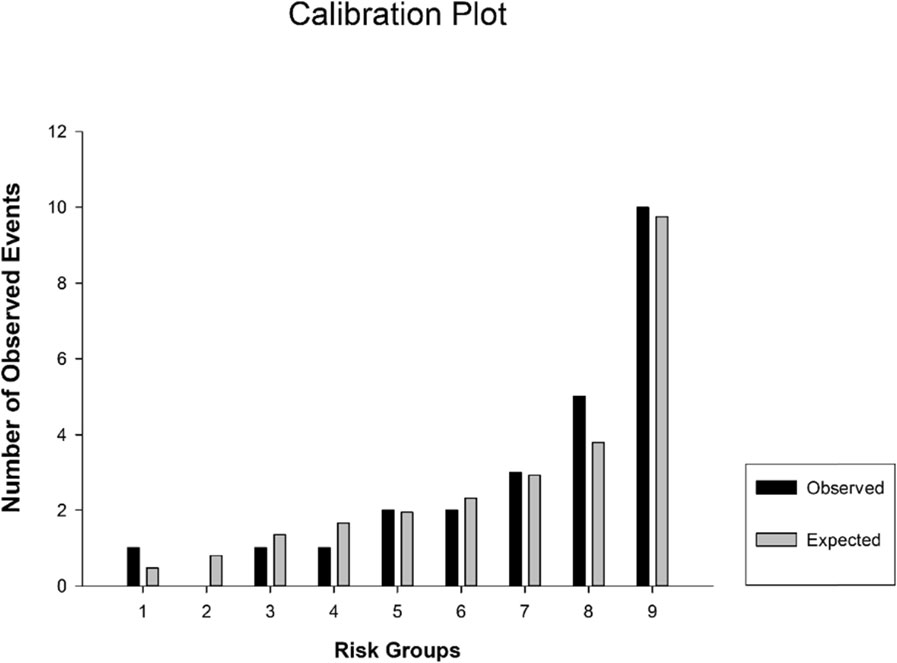
Calibration test of Log mtDNA+ Lac in predicting 28-d mortality

Since there was maximum overlap in the combined variable (log mtDNA + lactate) in septic patients and non-survivors, the ROC curve was analyzed (Table 3 and Fig. 1). Both plasma log mtDNA and lactate concentrations could effectively distinguish survivors and non-survivors in the ED (Table 3 and Fig. 2); however, the combined variable was the strongest predictor of mortality with area under the curve (AUC) values of 0.635–0.832 for lactate and 0.671–0.891 for log mtDNA, and moderate to high sensitivities and specificities (Table 3). The cut-off value for lactate was 2.29 (Youden’s index = 0.413) and was 5.01 (Youden’s index = 0.535) for log mtDNA (Table 3). For comparison, the AUC value for the combined variable was 0.698–0.901, with higher sensitivity and specificity, and the cut-off value was 5.46 (Table 3). The calibration test for the combined variable showed an X^2^ value of 2.559 and *P* = 0.923 (Fig. 3).

**Table 3:** Results of the ROC analysis of risk factors for sepsis prognosis

Variable	AUC	Cut-off(≥)	Sens.	Spec.	Youden’s index	LR+	LR-	*P* value	95%CI
Lac	0.724	2.29	0.80	0.613	0.413	2.067	0.326	0.001	0.635–0.832
Log mtDNA	0.781	5.01	0.76	0.775	0.535	3.378	0.310	0.001	0.671–0.891
LogmtDNA+Lac	0.799	5.46	0.72	0.80	0.520	3.60	0.35	0.001	0.698–0.901

## Discussion

We investigated plasma mtDNA levels in ED patients admitted with sepsis or septic shock, which were significantly higher in the septic shock group and correlated with the SOFA score and sepsis 28-d mortality. Moreover, the mtDNA levels showed superior prognostic prediction value than that of lactate levels.

Based on the sepsis 3.0 definition, a poor prognosis of sepsis is associated with organ injury, which is in line with the positive correlation of plasma mtDNA levels and severity of illness or SOFA score, even after controlling for potential confounders. We chose to focus our study on the level of free mtDNA in the plasma based on the observations by Zhang et al. [11] suggesting that mtDNA directly induces inflammation due to homology with pathogen-associated molecular patterns, thus acting as a DAMP. Our results are also consistent with those of Nakahira et al. [14] who conducted one of the first clinical trials on this topic in 2013, demonstrating significantly higher mtDNA levels in patients who died within 28 days of ICU admission than those of the survivors.

Moreover, mtDNA has been shown to improve risk prediction compared to that of commonly measured biomarkers such as lactate and PCT. In 2015, Bhagirath et al. [17] published a translational study aimed at elucidating the role of nuclear DNA (nucDNA), mtDNA, and bacterial DNA in sepsis. They showed that the levels of plasma nucDNA and mtDNA were 200- and 50-fold greater in the patients with sepsis than those of healthy controls, respectively. Timmermans et al. [18] conducted a prospective observational study by collecting samples from 121 septic shock patients in the ICU on day 1, 3, 5, 6, 9, 14, 21, and 28, demonstrating that the levels of nucDNA and mtDNA were significantly elevated and remained elevated at all time points relative to those of healthy controls. Nevertheless nucDNA, but not mtDNA, levels were associated with mortality from septic shock, which is inconsistent with our findings. We speculate that these differences are due to variations in the study population and diagnostic criteria between studies. Kung et al. [19] demonstrated that both plasma nucDNA and mtDNA concentrations on admission were significantly higher in non-survivors than in survivors, and both levels increased shortly after severe infection and then gradually decreased after antimicrobial therapy. Moreover, the nucDNA levels were significantly higher than the mtDNA levels for the same group. In a recent study, Yang et al. [20] indicated that the relative mononuclear cell mtDNA copy number in non-survivors was significantly lower than that of survivors, which was also an important predictor of clinical outcome, as patients with low copy numbers had higher 28-d mortality rates. This suggests that the nucDNA was first elevated in the initial stages of sepsis, accompanied by a depletion of mtDNA copy numbers, followed by the release of mtDNA in the plasma becoming DNA fragments, or mtDNA DAMPs, which are associated with the susceptibility and pathogenesis of sepsis-associated organ injury [21, 22]. The potential contribution of mtDNA DAMPs to organ injury is also supported by persuasive evidence from cell culture and animal models in which administration of exogenous mtDNA fragments or prevention of their accumulation had concordant effects on cytotoxicity, cellular dysfunction, and tissue inflammation [11, 23–27].

Innocenti et al. [28] demonstrated that higher lactate levels and decreased clearance were associated with increased short-term and intermediate-term mortality in patients with sepsis, regardless of the presence of shock. Timmermans et al. [18] also suggested that the plasma mtDNA level on admission was a more powerful predictor than lactate concentration, which is more commonly used for outcome prediction in clinical practice. This conclusion is consistent with our present findings. Therefore, we hypothesized that the plasma mtDNA might become elevated at an earlier stage than serum lactate; however, further prospective study is needed to verify this possibility.

### Limitations

Several limitations of the study deserve consideration. First, we only measured plasma levels on admission in the ED and did not measure levels serially, and therefore could not assess the variation of these levels in the ICU or over time. Second, large-scale prospective studies are warranted to evaluate the prognostic contribution of plasma DNA on clinical outcomes. Third, we did not measure intracellular mtDNA levels, and thus cannot extrapolate these findings to the variation in cellular mtDNA content. Finally, we did not examine the mtDNA levels of healthy controls for comparison owing to limitations of funding and ethical considerations.

### Conclusions

Plasma mtDNA levels were associated with the SOFA score and a poor prognosis of patients with sepsis in the emergency room. Plasma mtDNA could serve as a predictor of 28-d mortality of patients with sepsis in the ED.

## Data Availability

The datasets used during the current study are available from the corresponding author on reasonable request.

## Abbreviations

mtDNA: Mitochondrial DNA
ICU: Intensive care unit
DAMP: Damage-associated molecular pattern
ED: Emergency department
SOFA: Sequential Organ Failure Assessment
SCM: Septic cardiomyopathy
BNP: B-type natriuretic peptide
qPCR: Quantitative real-time polymerase chain reaction
RSTI: Respiratory tract infection
CHD: Coronary heart disease
ROC: Receiver operating characteristic
CRP: C-reactive protein
PCT: Procalcitonin
AUC: Area under the curve
nucDNA: Nuclear DNA
